# Neutralization effect of plasma from vaccinated COVID-19 convalescents on SARS-CoV-2 Omicron variants

**DOI:** 10.3389/fimmu.2022.975533

**Published:** 2022-09-29

**Authors:** Yudi Xie, Lei Liu, Jue Wang, Yaqiong Zheng, Chen Luo, Wenxu Ni, Zhihang He, Xin Zhao, Yan Liu, Yingyu He, Shangen Zheng, Ling Li, Zhong Liu

**Affiliations:** ^1^ Institute of Blood Transfusion, Chinese Academy of Medical Sciences and Peking Union Medical College, Chengdu, China; ^2^ Key Laboratory of Transfusion Adverse Reactions, Chinese Academy of Medical Sciences (CAMS), Chengdu, China; ^3^ General Hospital of Central Theater Command of the PLA, Wuhan, China; ^4^ College of Public Hygiene of Anhui Medical University, Hefei, China

**Keywords:** SARS-CoV-2, convalescent, vaccine, neutralization, delta, omicron

## Abstract

**Background:**

COVID-19 has caused a global pandemic and the death toll is increasing. With the coronavirus continuously mutating, Omicron has replaced Delta as the most widely reported variant in the world. Studies have shown that the plasma of some vaccinated people does not neutralize the Omicron variant. However, further studies are needed to determine whether plasma neutralizes Omicron after one- or two-dose vaccine in patients who have recovered from infection with the original strain.

**Methods:**

The pseudovirus neutralization assays were performed on 64 plasma samples of convalescent COVID-19 patients, which were divided into pre-vaccination group, one-dose vaccinated group and two-dose vaccinated group.

**Results:**

In the three groups, there were significant reductions of sera neutralizing activity from WT to Delta variant (B.1.617.2), and from WT to Omicron variant (B.1.1.529) (ps<0.001), but the difference between Delta and Omicron variants were not significant (p>0.05). The average neutralization of the Omicron variant showed a significant difference between pre-vaccination and two-dose vaccinated convalescent individuals (p<0.01).

**Conclusions:**

Among the 64 plasma samples of COVID-19 convalescents, whether vaccinated or not, Omicron (B.1.1.529) escaped the neutralizing antibodies, with a significantly decreased neutralization activity compared to WT. And two-dose of vaccine could significantly raise the average neutralization of Omicron in convalescent individuals.

## Introduction

In the past two years, the COVID-19 pandemic has produced wave after wave of SARS-CoV-2 mutants, which beat the early variants in showing partial resistance to neutralizing antibodies induced by natural infection and vaccination. The earliest mutants carried unimodal mutation D614G, which provided an advantage for transmission, and quickly replaced the ancestral virus as the main pandemic variant before May 2020 (1). By early 2021, the Alpha variant has come close to dominance, but would soon be overtaken by the Delta variant, which has dominated the pandemic since mid-2021. Alpha and Delta were modest neutralization escape variants, being 2-3 folds less susceptible than D614G to neutralization by mRNA-1273 vaccine-induced antibodies (2). However, these variants had little effect on the efficacy of mRNA-1273 vaccine (3).

The newly emerged SARS-CoV-2 Omicron variant, which was first discovered in South Africa in November 2021, presents a different scenario and causes major concerns (4, 5). It was designated the variant B.1.1.529 by World Health Organization (WHO) on the 26th of November 2021 (5). Since then, Omicron has spread globally (6). This variant appears to be more infectious than Delta, which has already caused super-spreader events (7) and has outcompeted Delta within weeks in some countries and metropolitan areas (6). Because there are many spike mutations in Omicron, including 15 mutations in the receptor binding domain (RBD), which is the main target of neutralizing antibodies, it has led to a reduction in the sensitivity of neutralizing antibodies. In SARS-CoV-2 convalescent or vaccinated individuals, the number of neutralizing epitopes targeted by polyclonal antibodies is an important determinant of the genetic barrier of virus escape (8). There are many antibody targets in SARS-CoV-2 spike proteins, but the polyclonal neutralization reaction is mainly dominated by antibodies against spike RBD and N-terminal domain (NTD) (8–11).

Wilfredo et al. evaluated the neutralizing immune effect of antibodies induced by mRNA-1273 and BNT162b vaccines against SARS-CoV-2 Omicron mutants and detected no neutralizing antibodies against Omicron in most vaccinated people (12). However, for patients who have recovered from infection with the original strain, further studies are needed to determine whether their plasma has a neutralizing effect against Omicron after one- or two-dose vaccine. In this study, we evaluated the neutralization of plasma samples which were collected from convalescent COVID-19 patients in Wuhan City, which had received either one or two doses of vaccine. We aimed to explore the protective effects of vaccination against Omicron in convalescents infected with the original strain, given that convalescent plasma with high neutralizing activity may be a source of isolating and developing useful monoclonal antibodies (mAbs) for the clinical treatment of novel coronavirus mutants.

## Methods

### Ethics statement

The study was approved by the Ethics Committee of the Institute of Blood Transfusion, Chinese Academy of Medical Sciences & Peking Union Medical College. All participants provided written informed consent to collecting of information, and the data generated by the study has been agreed to be published.

### Participants

**Figure 1:**
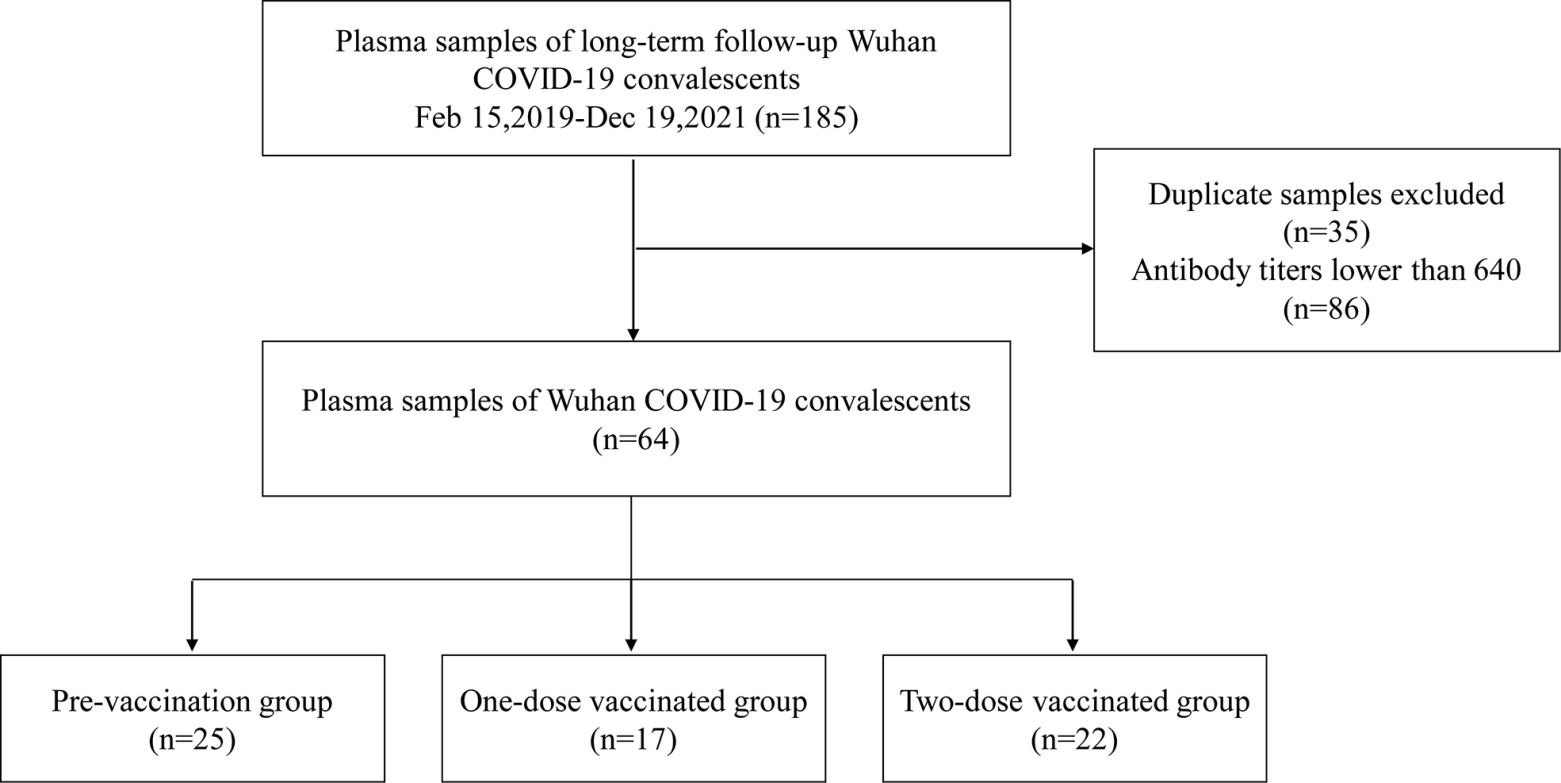
Study population flow diagram.

We collected 185 plasma samples of COVID-19 convalescents in Wuhan during Feb 15 to Dec 19, 2021. 35 duplicate samples from the same participants with the same times of vaccinations and 86 samples with antibody titers less than 640 were excluded. Thus, 64 plasma samples were finally included. The samples were divided into three groups——the pre-vaccination group, one-dose vaccinated group and two-dose vaccinated group, as shown in **Figure 1**. Except for one participant in the one-dose vaccinated group who received adenovirus vaccine (CanSinoBIO), all other participants received inactivated virus vaccine (Sinopharm or Sinovac).

### ELISA

Antibody titer was detected using the WANTAI SARS-CoV-2 RBD Antibody Detection Kit (Beijing Wantai Biological Pharmacy Ent., Lot: NCOG20210702B) according to the instructions. Microporous plates were precoated with SARS-CoV-2 RBD protein. The samples were diluted and added to microporous plates and incubated at 37°C for 1h. The microporous plates were washed for five times. The horseradish peroxidase labeled mouse-anti-human IgG monoclonal antibody was added and incubated at 37°C for 30mins. After incubation, the plate was washed five times, substrate buffer containing hydrogen peroxide and TMB was added to the wells, incubating at room temperature for 15mins. Then the termination solution was added, and a spectrophotometer was used to detect the OD value of the wells under dual-wavelength excitation light of 450nm and 630nm. The antibody titer was obtained by comparing the OD value with the standard substance, which was a monoclonal antibody with a defined titer (1280) and provided in the kit.

### Vero cell culture

Vero cells (provided by Nanjing Vazyme Biotechnology Co.) derived from the kidneys of normal adult African green monkeys that stably expressing endogenous ACE2. Cells were cultured with DMEM (1%(v/v) antibiotics, 10% (v/v) FBS) at 37°C, supplied with 5% CO_2_.

### Pseudovirus neutralization assay

The pseudovirus neutralization assays were performed using Vero cells. Various concentrations of plasma samples (3-fold serial dilution using DMEM) were transferred into 96-well white flat-bottom culture plates, mixed with SARS-CoV-2 pseudoviruses [SARS-CoV-2-Fluc WT (Nanjing Vazyme Biotechnology Co., Lot: 7E501L1), SARS-CoV-2-Fluc B.1.617.2 (Nanjing Vazyme Biotechnology Co., Lot: 7E501k1), SARS-CoV-2-Fluc B.1.1.529 (Nanjing Vazyme Biotechnology Co., Lot: 7E551L1)], and incubated for 1h at 37°C, supplied with 5% CO_2_. Plasma from healthy donors before the COVID-19 outbreak were included as negative controls. And negative control wells were supplied with 100μL DMEM (1%(v/v) antibiotics, 10% (v/v) FBS). Positive control (Nanjing Vazyme Biotechnology Co., Lot: 7E551K1) wells were supplied with 100μL DMEM. Pre-mixed Vero cells (100μL, 5×10^4^ in DMEM) were added to all wells, and the 96-well plates were incubated for 24h at 37°C, supplied with 5% CO_2_. After the incubation, 150μL of supernatants were removed, and 100μL Bio-Lite Luciferase reagent (Nanjing Vazyme Biotechnology Co., Lot: 7E531k1) was added to each well and incubated for 3 mins. After the incubation, luciferase activity was measured using a microplate spectrophotometer. The neutralization titer was calculated by comparing the OD value to the negative and positive control wells.

### Statistical analysis

Statistical analysis was conducted using GraphPad Prism 8 and SPSS 25. All data in the figures were presented as mean ± SD. The age and gender of three groups were evaluated by chi-square test. Differences of sera neutralizing activity among 3 groups and participants with different times of vaccination were evaluated by one-way ANOVA test. P < 0.05 was considered significant (*p < 0.05, **p < 0.01, and ***p < 0.001).

## Results

### Basic characteristics of the participants

We included samples from pre-vaccination convalescent individuals (n=25), one-dose vaccinated convalescent individuals (n=17) and two-dose vaccinated convalescent individuals (n=22). All the samples were part of a long-term follow-up COVID-19 convalescents cohort.

The median age of the three groups were 37 (range 26-68 years), 45 (range 31-70 years) and 44.5 (range 29-65 years). There were no differences among the 3 groups, neither in age nor in gender (**Table 1**).

**Table 1:** Basic characteristics of participants from three cohorts, including age, gender, time since onset of symptoms and time since last vaccination.

	Pre-vaccination group (n = 25)	One-dose vaccinated group (n = 17)	Two-dose vaccinated group (n = 22)	P value
**Age (years), median (range)**	37 (26-68)	45 (31-70)	44.5 (29-65)	0.138
**Gender**				0.617
Male, n (%)	15 (60.0%)	8 (47.1%)	11 (50.0%)	
Female, n (%)	10 (40.0%)	9 (52.9%)	11 (50.0%)	
**Time since onset of symptoms (month), mean (SD)**	9.221 (7.530)	17.83 (2.048)	19.20 (6.674)	
**Time since last vaccination (month), mean (SD)**	**-**	1.741 (1.141)	1.518 (0.753)	

### RBD IgG antibody titer and sera neutralizing activity

**Figure 2:**
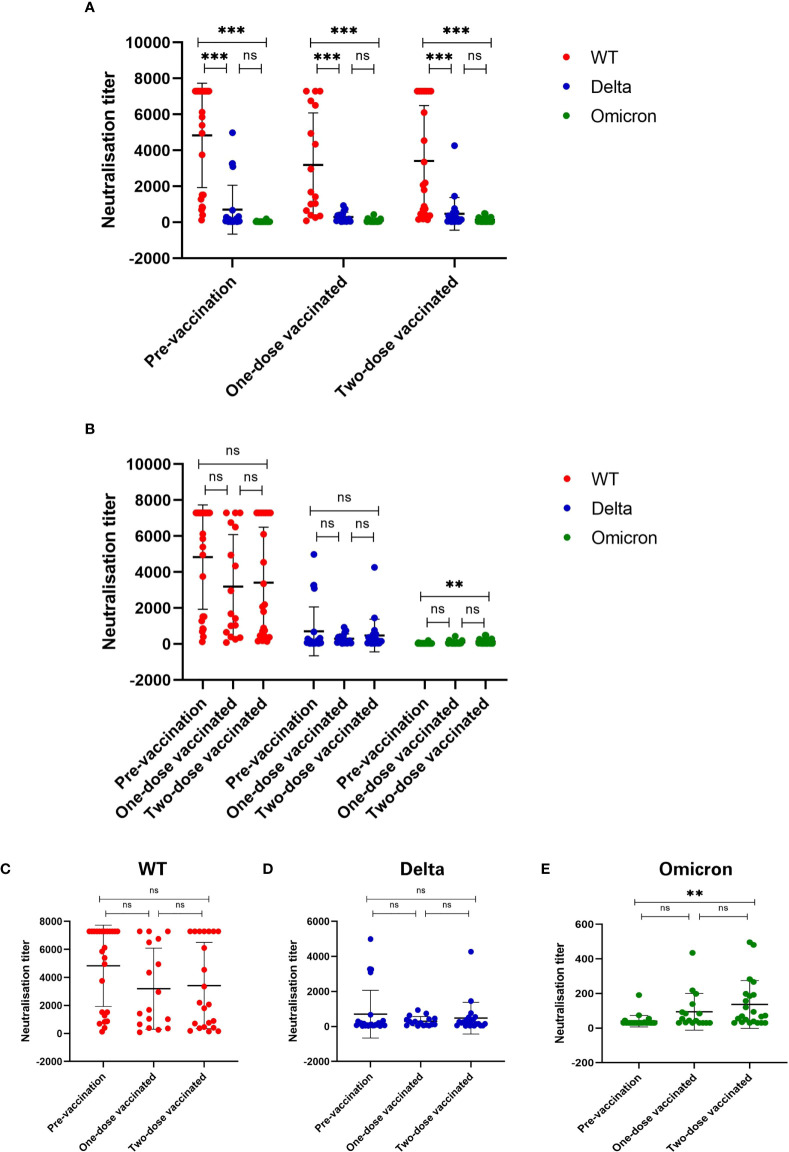
Sera neutralizing activity against three types of SARS-CoV-2 virus. **(A)** Sera neutralizing activity has been significantly reduced against Delta and Omicron as compared to WT SARS-CoV-2 (p < 0.001). **(B)** Comparison of sera neutralizing titers among three groups. **(C-E)** Sera neutralizing activity against **(C)** WT, **(D)** Delta (B.1.617.2) and **(E)** Omicron (B.1.1.529). Sera neutralizing activity was significantly enhanced after two-dose of vaccine as compared to before vaccination (p < 0.01). *p < 0.05, **p < 0.01, ***p < 0.001, and ns, no significance.

Compared to the OD value with the standard substance, antibody titers of all 64 plasma samples were 640 or higher (**Table 2**). Then we conducted the pseudovirus neutralization assays in VERO cells to compare the sera neutralizing activity against SARS-CoV-2 WT, Delta variant, and Omicron variant (**Figure 2**). In all three groups, we observed a significant reduction of sera neutralizing activity from WT to Delta variant (p<0.001), and from WT to Omicron variant (p<0.001, **Figure 2A**). However, the difference between Delta and Omicron variants in pre-vaccination group was not significant, and it was the same in one- and two-dose vaccinated groups (ps>0.05, **Figure 2A**). Notably, the average neutralization against Omicron variant showed a significant increase between pre-vaccination and two-dose vaccinated convalescent individuals (p<0.01, **Figures 2B, E** **)**.

**Table 2:** RBD IgG antibody test results of participants among three groups.

	Pre-vaccination group (n = 25)	One-dose vaccinated group (n = 17)	Two-dose vaccinated group (n = 22)
**Titer, median (P25, P75)**	640 (640, 1280)	1280 (640, 1280)	1280 (640, 1280)
**1:200 diluted S/CO value, mean (SD)**	4.017 (2.312)	5.773 (5.321)	5.434 (3.835)

## Discussion

The new SARS-CoV-2 viral isolate Omicron (B.1.1.529) caused widespread concern because of its high transmissibility and ability to infect the previously exposed or vaccinated individuals (4, 5). In almost every region, Omicron cases outnumbered those of previous SARS-CoV-2 variants in a very short period (6).

In this study, we further divided the COVID-19 convalescents into various groups based on their vaccination status. We compared the neutralizing antibody titers after one and two doses of vaccine with that of pre-vaccination among COVID-19 convalescents for the first time. As a result, we found significantly reduced sera neutralizing activity from WT to Delta (B.1.617.2) variant, and from WT to Omicron (B.1.1.529) variant. This result was consistent with those of Wanwisa Dejnirattisai et al., Juan Manuel Carreño et al., Jingwen Ai et al. and Sandile Cele et al. (6, 13–15). Although there was numerous prior research on SARS-CoV-2 variants, only several of them included convalescents as participants. Juan Manuel Carreño’s research was a significant example (6), but it differed from our study in two ways. The first disparity was the type of vaccine. They were two types of mRNA vaccine in Carreño’s study while it was almost exclusively inactivated virus vaccine in this study. Secondly, they didn’t state which type of virus that their participants infected with, while it was clear that our participants infected with WT. An interesting finding in the current study is that the difference in neutralizing activity against Delta and Omicron variants in pre-vaccination group had no significance, and the results in one- and two-dose vaccinated groups were the same. This may be explained by the fact that the individuals of the plasma samples were Wuhan citizens who were infected with the wild-type virus in the early stage of the epidemic. Still, we could observe that there were a few plasma samples with high neutralizing activity against Omicron, even though the participants were vaccinated with vaccines based on the original SARS-CoV-2. This suggests that some antibodies raised from vaccinations can tolerate the mutations or these plasma samples have neutralizing antibodies against the conserved sites. In either side, such plasma samples may be a useful source to isolate and develop mAbs for the clinical treatment against new variants.

As the COVID-19 vaccination campaign moves forward, many COVID-19 convalescents had also been vaccinated. We had collected serum samples of such individuals. The results of pseudovirus neutralization assays showed that there was a significant difference between pre-vaccination and two-dose vaccinated convalescent individuals in terms of the neutralizing activity against the Omicron variant. The research of Wanwisa Dejnirattisai et al. (13) showed a similar result. They found that sera from vaccinated cases who had contracted the Delta variant showed higher neutralization to Omicron, compared with unvaccinated Delta-infected cases. Besides, an increased trend was observed for vaccinated convalescents in the average neutralizing activity to the WT virus, as well as to the Delta variant, although the differences were not statistically significant. One of the possible reasons could be the small sample size. The observation may be confirmed with a study with larger sample size. In addition, even though there was a significant increase in neutralization against Omicron, the average neutralizing activity was still low. This suggests Omicron leads to the escape from neutralizing antibody responses. Whether a vaccine based on the Omicron variant needs to be developed is likely to be the next concern.

Plasma from patients recovered from COVID-19 infection, namely convalescent plasma, is an explorative treatment with considerable historical evidence in other infectious diseases (16). Those convalescent plasma-relevant studies suggest that convalescent plasma with high antibody titers and/or neutralizing ability may reduce the viral load (17). Given such consideration, COVID-19 convalescents who have received two-dose vaccine may be superior donators for convalescent plasma.

There are several limitations to our study. These include the relatively small sample size which may not represent the neutralization titers in the population, the limited longitudinal data, the lack of data on persistence of neutralization antibody responses after two doses of vaccine, the lack of long-term follow-up, and the lack of live virus neutralization data. However, the current study design allows for a preliminary assessment of the effect of neutralizing antibodies against Omicron variant in convalescents after one and two doses of vaccine. Further studies are needed to confirm the finding.

## Conclusion

Among the 64 plasma samples of COVID-19 convalescents, whether vaccinated or not, there was a significant decrease in neutralization titer against two kinds of variants compared to WT, which means that Omicron (B.1.1.529) could escaped the neutralizing antibodies. Importantly, two-dose vaccine was found to raise the average neutralization against Omicron in convalescent individuals.

## Data availability statement

The original contributions presented in the study are included in the article/**Supplementary Material**. Further inquiries can be directed to the corresponding authors.

## Ethics statement

The studies involving human participants were reviewed and approved by the Ethics Committee of the Institute of Blood Transfusion, Chinese Academy of Medical Sciences & Peking Union Medical College. The patients/participants provided their written informed consent to participate in this study.

## Author contributions

YX, LeL and JW were responsible for plasma collection, antibody titer detection and manuscript-writing. YX also completed data collation and analysis. LiL and ZL contributed to study design, performing the research analysis, and revising the manuscript. LeL, JW and SZ were involved in the discussion about participant recruitment. CL, ZH and YL contacted the convalescents and confirmed their willingness to participate in this study. YZ, WN and YH collected the basic characteristics of the participants. All authors read and approved the final manuscript.

## Funding

This work was supported by the CAMS Innovation Fund for Medical Sciences (CIFMS) (2020-I2M-CoV19-006, 2021-1-I2M-060), Science & Technology Department of Sichuan Province (2020YFS0583) and the Natural Science Foundation of Hubei Province (2021CFB501).

## Acknowledgments

Our gratitude goes to the participants who contributed to this study and Vazyme which support us for the technical assistance.

## Conflict of interest

The authors declare that the research was conducted in the absence of any commercial or financial relationships that could be construed as a potential conflict of interest.

## Publisher’s note

All claims expressed in this article are solely those of the authors and do not necessarily represent those of their affiliated organizations, or those of the publisher, the editors and the reviewers. Any product that may be evaluated in this article, or claim that may be made by its manufacturer, is not guaranteed or endorsed by the publisher.

## Abbreviations

SARS-CoV-2, Severe acute respiratory syndrome coronavirus 2; COVID-19, Coronavirus disease 2019; WHO, World Health Organization; RBD, Receptor binding domain; NTD, N-terminal domain; mAb, Monoclonal antibody; ELISA, Enzyme-linked immunosorbent assay; TMB, Tetramethylbenzidine; OD, Optical density; ACE2, Angiotensin converting enzyme 2; DMEM, Dulbecco’s modified eagle medium; WT, Wild type.
